# Graphene/Polyaniline Aerogel with Superelasticity and High Capacitance as Highly Compression-Tolerant Supercapacitor Electrode

**DOI:** 10.1186/s11671-017-2395-z

**Published:** 2017-12-19

**Authors:** Peng Lv, Xun Tang, Ruilin Zheng, Xiaobo Ma, Kehan Yu, Wei Wei

**Affiliations:** School of Optoelectronic Engineering, Nanjing University of Post and Telecommunications, Nanjing, 210023 People’s Republic of China

**Keywords:** Superelasticity, Graphene aerogel, Polyaniline, Compression-tolerant, Supercapacitor

## Abstract

Superelastic graphene aerogel with ultra-high compressibility shows promising potential for compression-tolerant supercapacitor electrode. However, its specific capacitance is too low to meet the practical application. Herein, we deposited polyaniline (PANI) into the superelastic graphene aerogel to improve the capacitance while maintaining the superelasticity. Graphene/PANI aerogel with optimized PANI mass content of 63 wt% shows the improved specific capacitance of 713 F g^−1^ in the three-electrode system. And the graphene/PANI aerogel presents a high recoverable compressive strain of 90% due to the strong interaction between PANI and graphene. The all-solid-state supercapacitors were assembled to demonstrate the compression-tolerant ability of graphene/PANI electrodes. The gravimetric capacitance of graphene/PANI electrodes reaches 424 F g^−1^ and retains 96% even at 90% compressive strain. And a volumetric capacitance of 65.5 F cm^−3^ is achieved, which is much higher than that of other compressible composite electrodes. Furthermore, several compressible supercapacitors can be integrated and connected in series to enhance the overall output voltage, suggesting the potential to meet the practical application.

**Electronic supplementary material:**

The online version of this article (10.1186/s11671-017-2395-z) contains supplementary material, which is available to authorized users.

## Background

Rapid development of portable and wearable electronic devices not only enriches our daily lives, but also requires matchable energy storage devices, which should have the capability to endure high-level strains [1–3]. Among various strains, compression strain is one of the key factors that obviously affect the performances of energy storage devices [4, 5]. Supercapacitors (SCs) are the promising energy storage devices to power portable and wearable electronics due to their high power density, rapid charge rate and long cycle life [6, 7]. Recently, design and assembly of compression-tolerant SCs have attracted intense attentions. As one of the critical components in compression-tolerant SCs, electrodes are required to possess some features, such as mechanical robustness, resilient and durable. Carbon-based composite materials with sponge-like or foam-like structures have been studied as the compressible electrodes for compression-tolerant SCs (Table 1) [8–13]. However, these composite sponges or foams show recoverable compressive strains of only 50 ~ 75% (Table 1), which is not high enough to meet the practical application of compression-tolerant SCs.

**Table 1:** Comparison of the compressible composite electrodes. The capacitances are obtained with symmetric full cells

Materials	Uncompressed capacitance	Maximum compressive strain	Compressed capacitance	Content of pseudomaterials	Test condition	Ref.
Fe_2_O_3_/CNT sponge	296.3 F g^−1^	70%	266.7 F g^−1^	47 wt%	5 mV s^−1^	[8]
CNT@PPy sponge	376 F g^−1^	60%	338.4 F g^−1^	52.4 wt%	0.5 A g^−1^	[9]
CNT@ PPy@MnO_2_ sponge	305 F g^−1^ 9.6 F cm^−3^	50%	275 F g^−1^ 16.1 F cm^−3^	~ 60 wt%	2 mV s^−1^	[10]
Graphene/PPy foam	350 F g^−1^ 14 F cm^−3^	50%	350 F g^−1^ 28 F cm^−3^	N.A.	1.5 A g^−1^	[11]
Melamine foam/graphene/PPy sponge	411 F g^−1^	75%	329 F g^−1^	3.92 wt%	10 mV s^−1^	[12]
Sponge/PANI/CNT	216 F g^−1^ 3.4 F cm^−3^	60%	210 F g^−1^	65 wt%	0.64 A g^−1^	[13]
Graphene/PANI aerogel	424 F g^−1^ 8.9 F cm^−3^	90%	407 F g^−1^ 85.5 F cm^−3^	63 wt%	1 A g^−1^	This work

*NA* Not applicable

Superelastic graphene aerogels with ordered porous structure (such as honeycomb-like cellular structure [14, 15], bubble structure [16], and multi-arch structure [17]) show ultra-high compressibility (recoverable compressive strains reaching 90 ~ 99%). This ultra-high compressibility of the superelastic graphene aerogels arises from the integrated graphene pore walls and ordered porous structure [18, 19]. In the pore walls, the tightly integrated multilayered structure can maximize the π-π interaction between graphene sheets and thus greatly improve the strength of pore walls. And the pores organized in ordered structure provide the maximum elastic modulus for the graphene aerogels. There are already some reports about the application of superelastic graphene aerogels as the compressible electrodes of SCs [20, 21]. Although the maximum compressive strains of the superelastic graphene aerogel electrodes reach 90%, the specific capacitance of them (37 F g^−1^ [20], 90 F g^−1^ [21]) are still too low due to the double-layer storage mechanism of carbon materials.

To improve the specific capacitance of the graphene aerogel, an effect method is to combine the graphene aerogel with pseudocapacitive materials to form a composite aerogel electrode [7, 22]. For instance, Co_3_O_4_ [23], MnO_2_ [24, 25], polyaniline (PANI) [26], and polypyrrole (PPy) [27], have been introduced into the graphene aerogel to improve the electrochemical performances. For the study of the combination of superelastic graphene aerogels and pseusocapacitive materials, Zhao, et al. reported the compressible graphene/CNT/MnO_2_ aerogel as the electrodes of SCs [28]. However, the specific capacitance and recoverable compressive strains of the aerogel are too low (106 F g^−1^, strain = 50%). It is attributed to that the attachment of MnO_2_ particles on graphene/CNT scaffold is relative weak, and the mass content of MnO_2_ and compressive strains must be kept at low-level to avoid the peel-off of MnO_2_ from the scaffold.

Conducting polymer of PANI has been studied extensively as the electrode material due to its high conductivity, electroactivity, and specific pseudocapacitance [29]. And PANI can be well loaded on the surface of graphene due to the strong π-π interaction between the conjugated polymer and graphene [11, 13]. Herein, we introduced a new type of highly compression-tolerant electrode material with both high compressibility and high capacitance by depositing PANI into the superelastic graphene aerogel. In the graphene/PANI aerogels, the superelastic graphene aerogel as a conductive scaffold contributes its superelasticity and high electron conductivity. PANI deposited on the cell walls of the superelastic graphene aerogel produces high pseudocapacitance. And the strong interactions between PANI and graphene make the superelasticity of the graphene aerogel is well inherited after the deposition of PANI. We also fabricated the two-electrode all-solid-state SCs based on graphene/PANI electrodes to demonstrate their compression-tolerant ability. A gravimetric capacitance of 424 F g^−1^ is obtained and retains 96% even under 90% compression strain, allowing us achieve a high volumetric capacitance of 65.5 F cm^−3^.

## Methods/Experimental

### Preparation of Superelastic Graphene Aerogel

Graphene oxide (GO) was prepared by the oxidation of flake graphite according to the modified Hummers’ method [30, 31]. The superelastic graphene aerogel was fabricated using the ice template method [15]. In a typical procedure, GO aqueous dispersion (5 mg mL^−1^, 10 mL) was first mixed with L-ascorbic acid (100 mg) by stirring for 30 min. Then the mixture solution was poured into the glass vials and heated for 30 min at 90 °C for the synthesis of partially reduced graphene hydrogel. The obtained hydrogel was treated by the freeze-thaw process in the refrigerator (− 20 °C) and room temperature. Subsequently, further reduction process for the freeze-recast hydrogel was carried out for 5 h at 90 °C by the initial reductant (L-ascorbic acid) to get completely reduced graphene hydrogel. Finally, the graphene hydrogel was subjected to dialysis in deionized water and dried at 60 °C for 48 h to obtain the superelastic graphene aerogel.

### Preparation of Superelastic Graphene/PANI Aerogel

The electrochemical deposition of PANI into the superelastic graphene aerogel was carried out by cyclic voltammetry (CV) method using a three-electrode electrochemical workstation (CHI660E), where the superelastic graphene aerogel was used as the working electrode, a platinum electrode as the counter electrode, and a Ag/AgCl electrode as the reference electrode. The deposition process was performed in the potential range from − 0.2 to 0.8 V at a sweep rate of 50 mV s^−1^ for 100, 200, 300, and 400 cycles in 1 M H_2_SO_4_ and 0.05 M aniline aqueous solution. Subsequent to the electrochemical deposition, the samples were washed with deionized water and then dried at 60 °C for 24 h. The mass contents of PANI in graphene/PANI aerogels were calculated from the mass changes of the aerogels before and after electrochemical deposition. The graphene/PANI aerogels were defined based on the deposition period. For example, the graphene/PANI-2 aerogel was prepared by 200 CV sweeping cycles.

### Fabrication of Compressible All-Solid-State SCs

The compressible all-solid-state SCs were assembled to investigate the electrochemical performances of the graphene/PANI electrodes under various compressive strains. The assembly procedure has been mentioned in previous literatures [13, 32–34]. In a typical process, the PVA/H_2_SO_4_ gel electrolyte was first prepared through mixing H_2_SO_4_, PVA powder, and deionized water according to the mass ratio of 4:5:50. Subsequently, the mixture was stirred for 30 min at 80 °C to form a clear electrolyte. After that, the graphene/PANI aerogels were immersed into the PVA/H_2_SO_4_ gel electrolyte for 30 min and were curdled in the air. Then two pieces of the aerogels were placed onto two poly(ethylene terephthalate) (PET) substrates with Au (~ 100 nm), respectively. One piece of porous separator (Celgard 3501) was also infiltrated with PVA/H_2_SO_4_ gel electrolyte. The compressible all-solid-state SCs were obtained by assembling the as-prepared two electrodes sandwiched with the separator under pressure. Finally, the devices were kept at 45 °C for 24 h to remove excess water in the electrolyte.

### Materials Characterization

Micro-Raman spectroscopy (RM3000, Renishaw) was performed using a laser excitation wavelength of 514.5 nm. The microstructure of the graphene/PANI aerogels was observed using a Hatchi S-4800 scanning electron spectroscopy (SEM) equipped with energy dispersive spectroscopy (EDS). The chemical structure of the aerogels was investigated by Fourier transform infrared spectroscopy (FIIR, Nicolet 520) and X-ray photoelectron spectroscopy (XPS, PHI 1600 spectroscopy). Compression tests were carried out on an Instron-5566 with a strain rate of 100 mm min^−1^.

### Electrochemical Measurements

Electrochemical characterizations including CV, galvanostatic charge–discharge (GCD) and electrochemical impedance spectroscopy (EIS) were carried out by the CHI660E electrochemical workstation. Electrochemical measurements of the individual electrode were performed in a three-electrode system with 1 M H_2_SO_4_ aqueous electrolyte. The graphene/PANI aerogel, Pt wire, and Ag/AgCl were used as working electrode, counter electrode, and reference electrode, respectively. The specific capacitance (*C*_*s*_) was calculated from the GCD curves according to the following equation:

1 $$ {C}_s=I\times \varDelta t/m\times \varDelta V $$

where *I* is the constant discharge current, *∆t* is the discharging time, *m* is the mass of the working electrode, *∆V* is the voltage drop upon discharging.

The electrochemical measurements of the electrodes under various compressive strains were performed in the all-solid-state SCs in the original state or at certain compressive strains. The gravimetric capacitance (*C*_*g*_) and volumetric capacitance (*C*_Vol_) of the graphene/PANI electrodes in the SCs were calculated from the GCD curves according to the following formulas:

2 $$ {C}_g=4\times I\times \varDelta t/m\times \varDelta V $$

3 $$ {C}_{\mathrm{Vol}}=\rho \times {C}_g $$

where *I* is the constant discharge current, *∆t* is the discharging time, *m* is the total mass of two electrodes, *∆V* is the voltage drop upon discharging, *ρ* is the density of the graphene/PANI aerogel under various compressive strains.

The energy density (*E*) and power density (*P*) of the SCs were calculated from the GCD curves using the following equations.

4 $$ E={C}_g\times \varDelta {V}^2/8\times 3.6 $$

5 $$ P=3600\times E/\varDelta t $$

## Results and Discussion

**Fig. 1:**
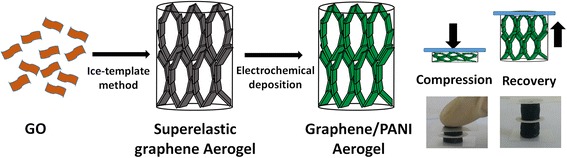
Illustration for the fabrication process of compressible graphene/PANI aerogel

**Fig. 2:**
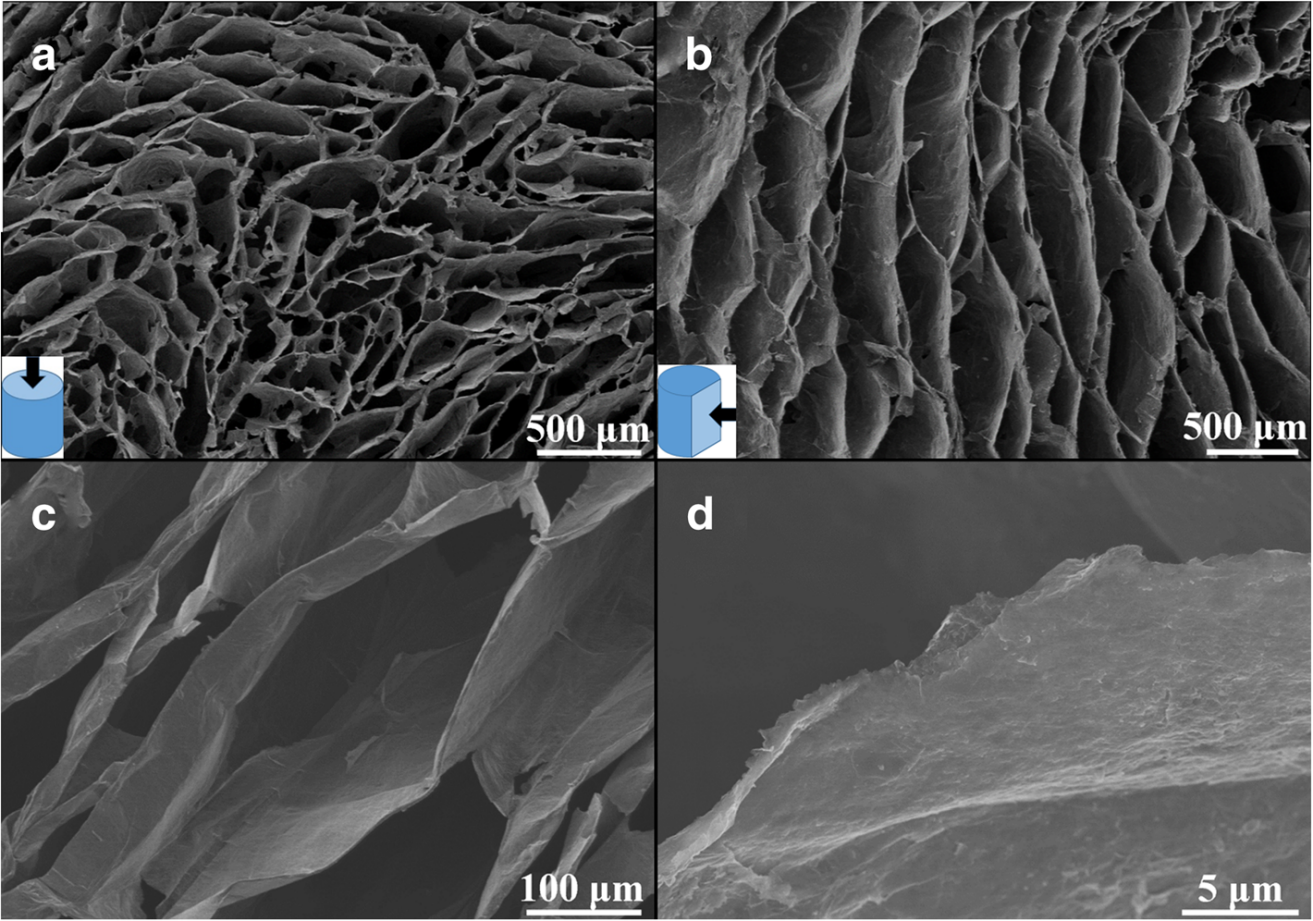
SEM images of **a** cross-section and **b** vertical-section of the superelastic graphene aerogel. **c**, **d** Cross-section view of superelastic graphene aerogel at different magnifications

The fabrication processes of the compressible graphene/PANI aerogel are illustrated in Fig. 1. The superelastic graphene aerogel is assembled from the GO aqueous solution using the ice-template method and subsequent reduction process [15]. Then the PANI is deposited onto the cell walls of the as-prepared superelastic graphene aerogel by electrochemical deposition method. The structural change of GO before and after the reduction processes is reflected by Raman spectra (Additional file 1: Fig. S1). It indicates that reduction processes remove partial oxygen containing functional groups of GO, which would provide strong π-π interaction between graphene sheets. The microstructure of the superelastic graphene aerogel was observed by SEM. As shown in Fig. 2a, b, the superelastic graphene aerogel presents the highly porous, honeycomb-like, and oriented cellular structure at both cross-section view and vertical-section view. The graphene sheets are closely packed and well oriented in parallel manner to form cell walls of the graphene aerogel (Fig. 2c, d). Those honeycomb-like structure and oriented cells enhance the mechanical robustness of cell walls and bring the graphene aerogel superelasticity, which is also mentioned in previous literatures [15, 35–37]. It is noteworthy that the cell dimension of the graphene aerogel is about hundreds of micrometers due to relative low freezing rate during the recasting process. This huge cell dimension is in favor of the impregnation of aniline monomer solution and uniform distribution of PANI during the electrochemical deposition process.

**Fig. 3:**
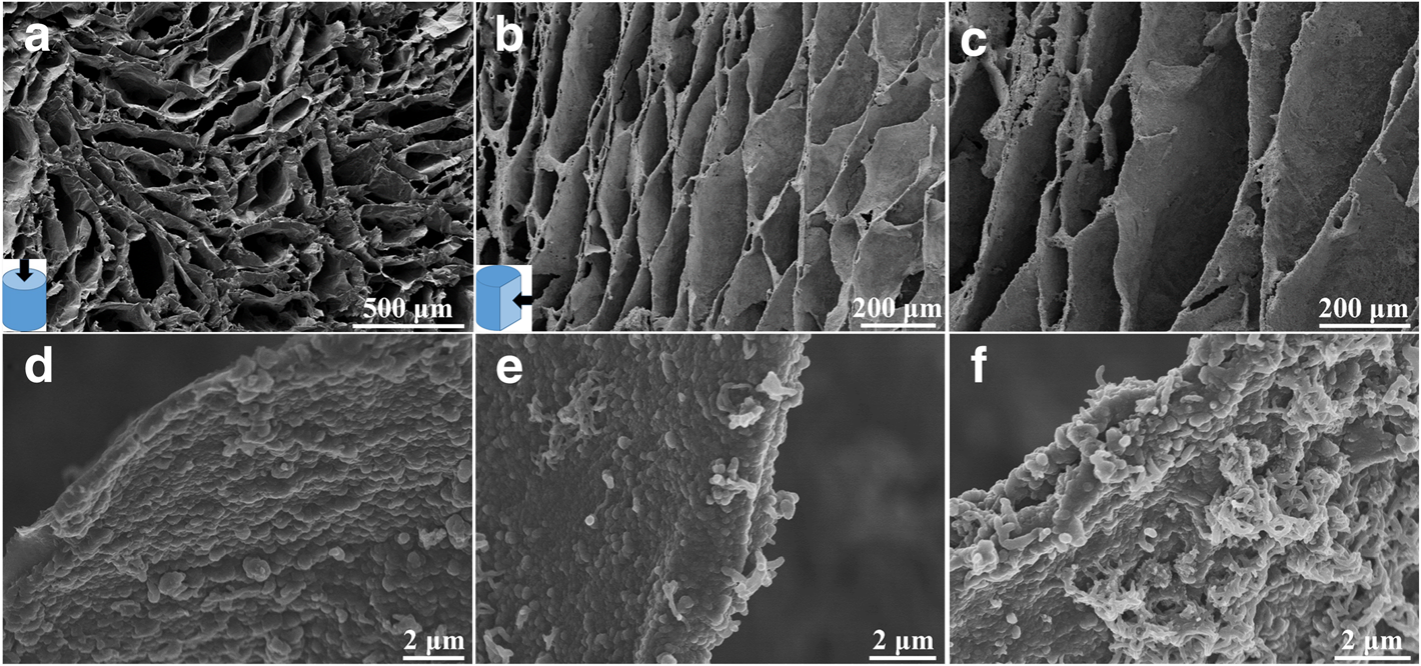
SEM images of **a** cross-section and **b**, **c** vertical-section of graphene/PANI-2 aerogel. **d** SEM images of graphene/PANI-1. **e** Graphene/PANI-2. **f** Graphene/PANI-3 aerogels at high magnifications

After electrochemical deposition process, the microstructure of the graphene/PANI aerogels was observed. As shown in Fig. 3a–c, the highly porous, honeycomb-like, and oriented cellular structure of the superelastic graphene aerogel is well inherited without any collapse after the deposition process. As displayed in the SEM images of graphene/PANI-1 and graphene/PANI-2 aerogels at high magnification (Fig. 3d, e), it can be found that plenty of PANI nanocones homogeneously and erectly grow on the whole surface of graphene cell walls, which is significantly different from the smooth surface of the cell walls in superelastic graphene aerogel (Fig. 2d). This nanocone surface coating is similar to the PANI layer deposited in the 3D graphene aerogel [38] or on the porous carbon nanofibers [39]. The large-area cross-section and vertical-section SEM (Fig. 3a, b) and the EDS element mapping (Additional file 1: Fig. S2) show the homogeneous distribution and conformal coating of PANI throughout the interior zone of superelastic graphene aerogel, which is attributed to that macroporous structure and large cell dimension of superelastic graphene aerogel enable the fast flux and uniform penetration of precursor into the interior zone of the superelastic graphene aerogel. Furthermore, the mass content of PANI in graphene/PANI aerogels can be well controlled by the deposition period (Additional file 1: Table S1). Figure 3d–f also shows the morphology evolution of PANI nanocones corresponding to different CV sweeping cycles. The thickness of PANI nanocone layer gradually increases with the increase of deposition period. When the CV sweeping cycles reach 300, the PANI coating on graphene cell walls become non-uniform and non-conformal (Fig. 3f). Overdepositing of PANI results in the formation of PANI nanowire network on the outer layers of graphene cell walls. When the deposition cycles reach 400, the nanowire network covered the whole surface of the cell walls (Additional file 1: Fig. S3), however, they are easily washed away with water.

**Fig. 4:**
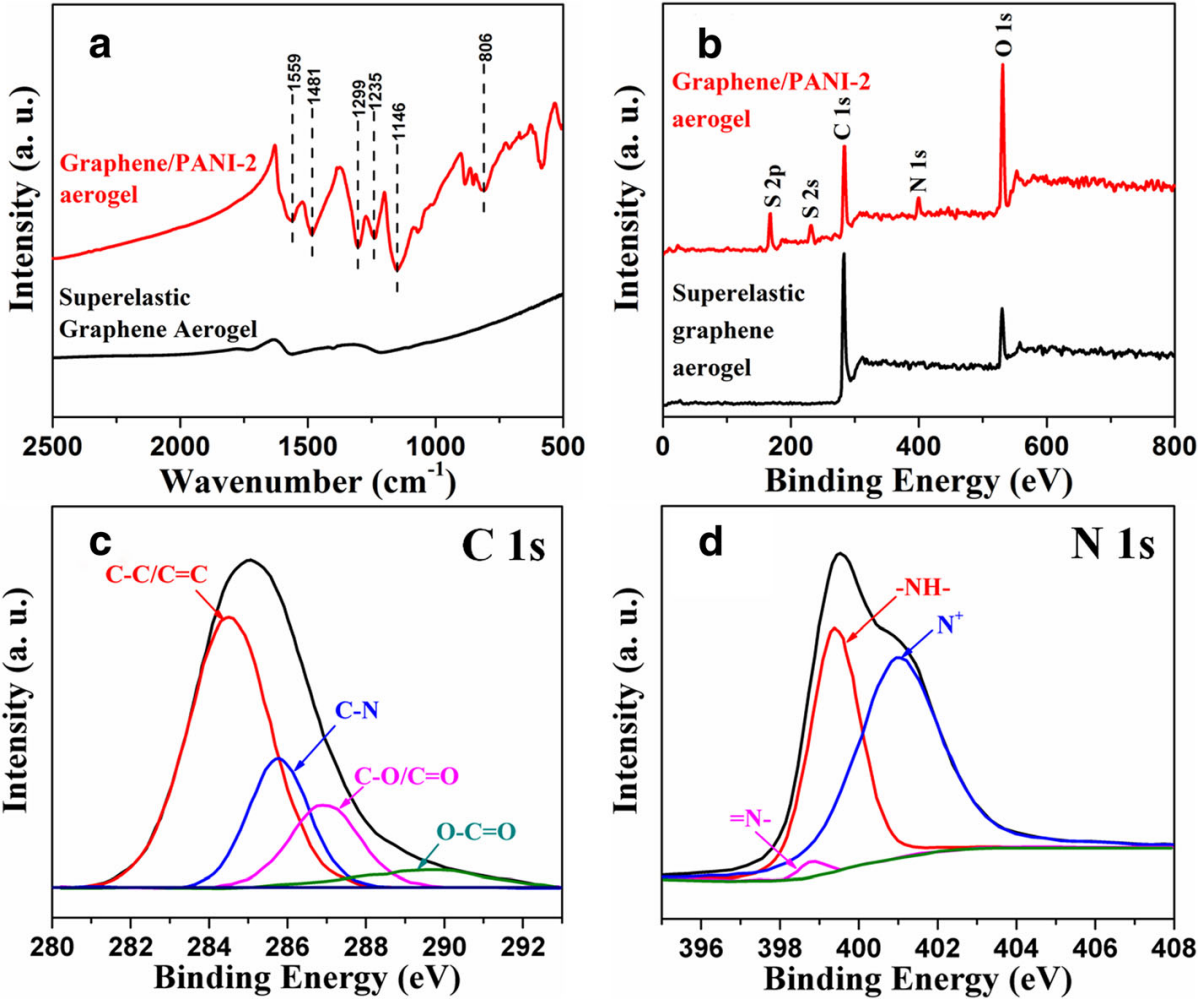
**a** FTIR spectra and **b** XPS spectra of superelastic graphene aerogel and graphene/PANI-2 aerogel. **c** C 1 s and **d** N 1s spectra of graphene/PANI-2 aerogel

In order to reveal the chemical structure of the graphene/PANI aerogels, the FTIR spectrum of graphene/PANI-2 aerogel is shown in Fig. 4a. The peaks at 1559 and 1481 cm^−1^ correspond to the C═C stretching of quinoid ring and benzenoid ring. The peaks at 1299 and 1235 cm^−1^ correspond to C─N stretching vibrations with aromatic conjugation. The peaks at 1146 and 806 cm^−1^ correspond to in-plane and out-of-plane bending vibration of C─H [26, 40, 41]. XPS was further performed to characterize the composition of graphene/PANI-2 aerogel (Fig. 4b). In comparison of superelastic graphene aerogel, graphene/PANI-2 aerogel presents the additional N 1s peak and S 2p peak besides the O 1s and C 1s peaks, confirming the existence of PANI and that PANI is doped by SO_4_^2−^ [26, 38]. The C 1s spectrum (Fig. 4c) contains four peaks of C─C/C═C, C─N, C─O/C═O, and O─C═O at 284.4, 285.6, 286.6, and 290.2 eV, respectively [42]. The deconvolution of N 1s core-level spectrum (Fig. 4d) results in three peaks ascribed to PANI: quinoid imine (─N═), benzenoid amine (─NH─), and positively nitrogen cationic radical (N^+^) at 398.8, 399.3, and 401.1 eV, respectively [42, 43]. The last peak is indicative of the doped state of PANI in the composite. The high ratio of N^+^ illustrates a high proton doping level for the deposited PANI on graphene cell walls, leading to the enhancement of electron conductivity and pseudocapacitive performance. Additional file 1: Fig. S4 shows the XRD patterns of superelastic graphene aerogel and graphene/PANI aerogels. The board peak of superelastic graphene aerogel appearing at 2θ = 26.2° corresponds to the (002) plane of graphitic phase, suggesting high extent of reduction [44]. Graphene/PANI aerogels present another intense crystalline peak mainly overlapped with graphitic phase peak at 2θ = 25.2°, corresponding to (002) planes of PANI [38, 41, 45]. In addition, the peak at 2θ = 19.6° (011) is also observed for graphene/PANI aerogels, which is decisive evidence indicating presence of PANI in the aerogels [38, 41, 45].

**Fig. 5:**
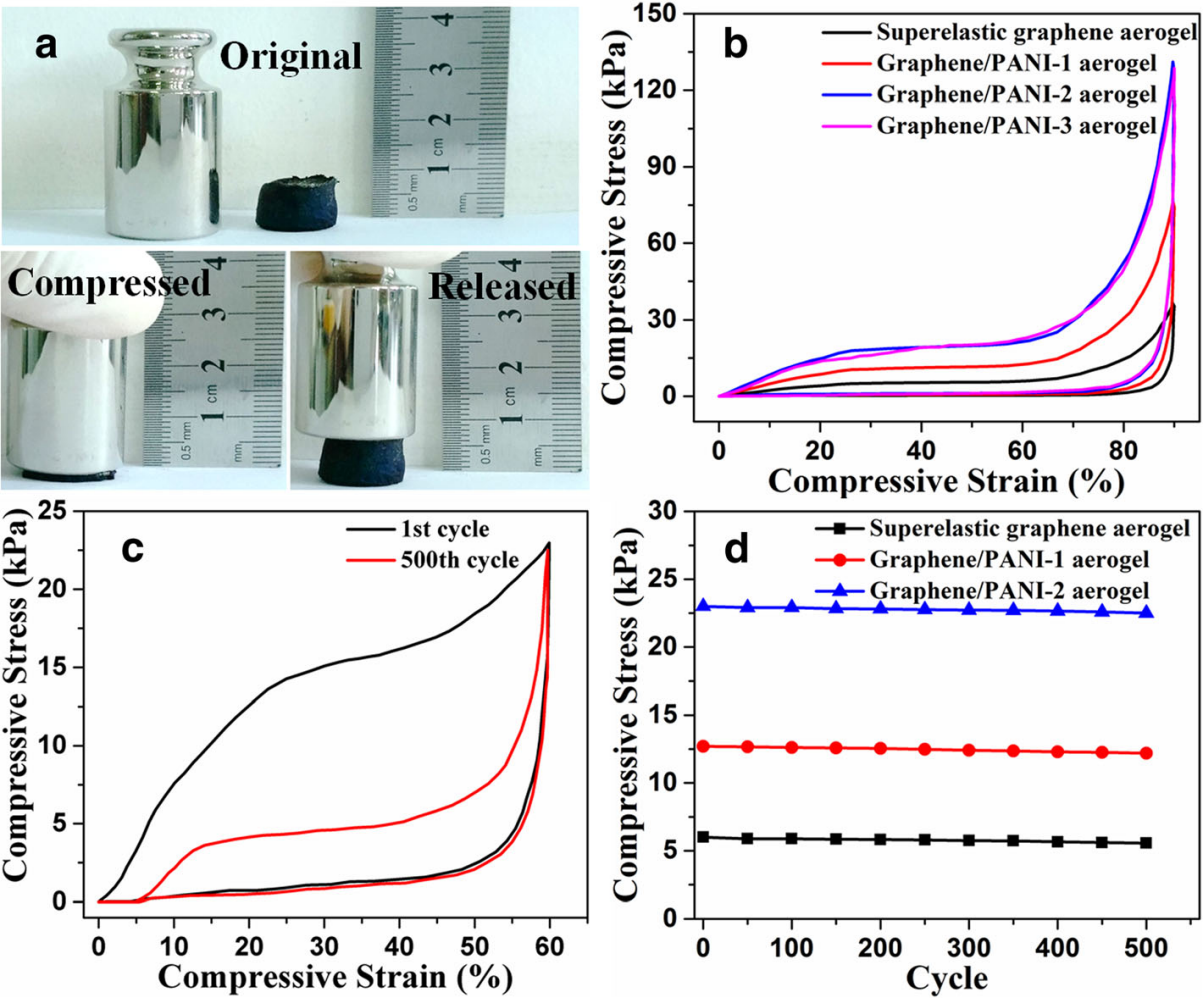
**a** Real-time photos of the compression-recovery process of graphene/PANI-2 aerogel. **b** Compressive stress–strain curves of superelastic graphene aerogel and graphene/PANI aerogels at a set strain of 90%. **c** Stress–strain curves of 1st and 500th cycles of graphene/PANI-2 aerogel at a set strain of 60%. **d** Maximum stress values of superelastic graphene aerogel and graphene/PANI aerogels for 500 cycles at a set strain of 60%

As mentioned in the previous reports, graphene aerogels with honeycomb-like and oriented cellular structure can present superelasticity [15, 46]. The uniaxial compression measurements of the graphene/PANI aerogels were also carried out to study the influence of the deposition of PANI on mechanical properties. As shown in Fig. 5a, graphene/PANI-2 aerogel can be squeezed into a pellet under manual compression and recover most of the volume without structural fatigue, indicating the high compressibility of graphene/PANI-2 aerogel. This compression-tolerant ability is also reflected by the inner microstructure of the graphene/PANI-2 aerogel during the compression-release process. The initial ordered cellar structure is conformably densified while keeping the continuous configuration under compression (Additional file 1: Fig. S5a). Once released, the graphene/PANI-2 aerogel rapidly recovers to the initial state without any collapse of the ordered cellar structure (Additional file 1: Fig. S5b). In addition, PANI nanocones are still tightly attached on the cell wall surface of superelastic graphene aerogel without obvious peel-off after the compression-release process (Additional file 1: Fig. S5c, d), indicating the strong interaction between graphene and PANI. The stress–strain curves of superelastic graphene aerogel and graphene/PANI aerogels are shown in Fig. 5b. For compressive strain up to 90%, the unloading curves all return to the origin without producing residual strain (plastic deformation). The maximum stress values of graphene/PANI-1~3 aerogels at strain of 90% range from 76 to 131 kPa, which is much higher than that of superelastic graphene aerogel (36 kPa). It indicates the strengthening effect of PANI coating for the superelastic graphene aerogel. Higher mass content of PANI results in thicker coating layer, making the whole network more rigid and resistant to compression. However, the stress values of graphene/PANI-3 aerogel are not higher than that of graphene/PANI-2 aerogel, which is attributed to that overdepositing of PANI leads the growth of PANI nanowire out of graphene sheets rather than coating on the cell wall surface. The cycle stability of elasticity for the graphene/PANI aerogels was also been measured. As shown in Fig. 5c, after 500 compression cycles at strain of 60%, graphene/PANI-2 aerogel develops a modest plastic deformation (residue strain of 5%). In addition, the graphene/PANI-2 aerogel can sustain the repeated compression cycles without significant stress degradation, indicating high structure stability (Fig. 5d). The maintaining of high compressibility and cycle stability after the deposition of PANI is attributed to the physical reinforcement of the graphene cell walls by the uniform coating of PANI. The PANI coating layers tightly adhere on the graphene cell walls due to the strong π-π interaction between PANI and graphene sheets. Upon loading, the load is effectively transferred between the graphene skeleton and the PANI coating layers. This unique structure can help to relax the local stress and dissipate the micro-crack energy. Similar mechanisms of 3D graphene reinforced by polymer have also been mentioned in previous literatures [10, 47].

**Fig. 6:**
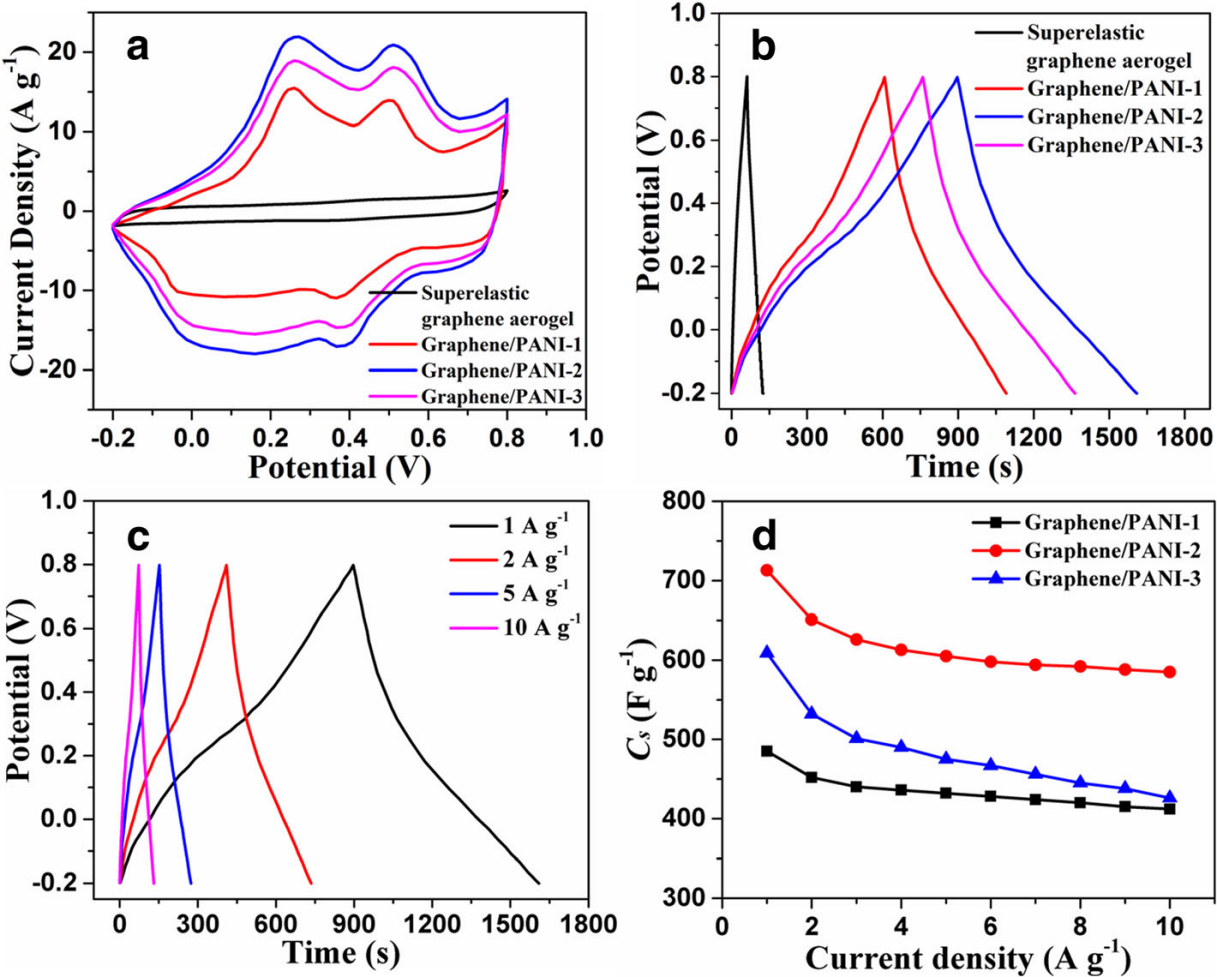
**a** CV curves and **b** GCD curves of superelastic graphene aerogel and graphene/PANI-1 ~ 3 aerogels, scan rate: 20 mV s^−1^, current density: 1 A g^−1^. **c** GCD curves and **d** specific capacitances of graphene/PANI-2 aerogel at different current density

The electrochemical performances of graphene/PANI aerogels were firstly investigated by CV and GCD tests using the three-electrode system in 1M H_2_SO_4_ aqueous solution. As shown in Fig. 6a, the functionalization of PANI in graphene/PANI aerogel causes higher current density and enlarged area than that of superelastic graphene aerogel, indicating the significant contribution of pseudocapacitance by PANI. Two couples of redox peaks are also observed from the CV curves of graphene/PANI aerogels, which is attributed to the leucoemeraldine/emeraldine and emeraldine/pernigraniline transitions of PANI [43, 48, 49]. Among all the graphene/PANI aerogels, graphene/PANI-2 aerogel possesses the largest area of surrounded CV loops, indicating an optimized mass content of PANI. Correspondingly, GCD curves of the graphene/PANI-1~3 aerogel at current density of 1 A g^−1^ are shown in Fig. 6b. In agreement with the CV results, the GCD curve of graphene/PANI-2 aerogel holds the highest discharge time and consequently highest specific capacitance (713 F g^−1^). This value of specific capacitance of the graphene/PANI-2 aerogel in this work is located at a moderate level among other 3D graphene/PANI composites in the previous reports (Additional file 1: Table S2). As discussed above, overdepositing of PANI leads to the unwanted growth of graphene nanowire out of graphene cell walls. In the case of graphene/PANI-3 aerogel, the graphene backbone cannot provide the reinforcement of conductivity and mechanical strength for the PANI nanowire due to the inferior contact between the PANI nanowire and graphene cell walls.

Figure 6c displays the GCD curves of graphene/PANI-2 aerogel at different current densities. The almost symmetric GCD curves indicate that the graphene/PANI aerogels possess a good capacitive behavior, where the deviation to linearity is typical of a pseudocapacitve contribution. The specific capacitances of the graphene/PANI-1~3 aerogels were calculated from the GCD curves at various current densities. As shown in Fig. 6d, graphene/PANI-2 aerogel shows higher specific capacitances than that of others. As the current density increases from 1 to 10 A g^−1^, the specific capacitance of graphene/PANI-2 aerogel has 82% retention of its initial value, indicating a good rate performance. The cycling stability of graphene/PANI-2 aerogel was tested along by repeating the GCD test at current density of 1 A g^−1^. As shown in Additional file 1: Fig. S7, its specific capacitance preserves 92% after 1000 cycles, showing an excellent cycling stability. In consideration of the good electrochemical performances of graphene/PANI-2 aerogel, the subsequent researches of the compressible electrodes in compression-tolerant SCs in this work were all based on the graphene/PANI-2 electrodes.

**Fig. 7:**
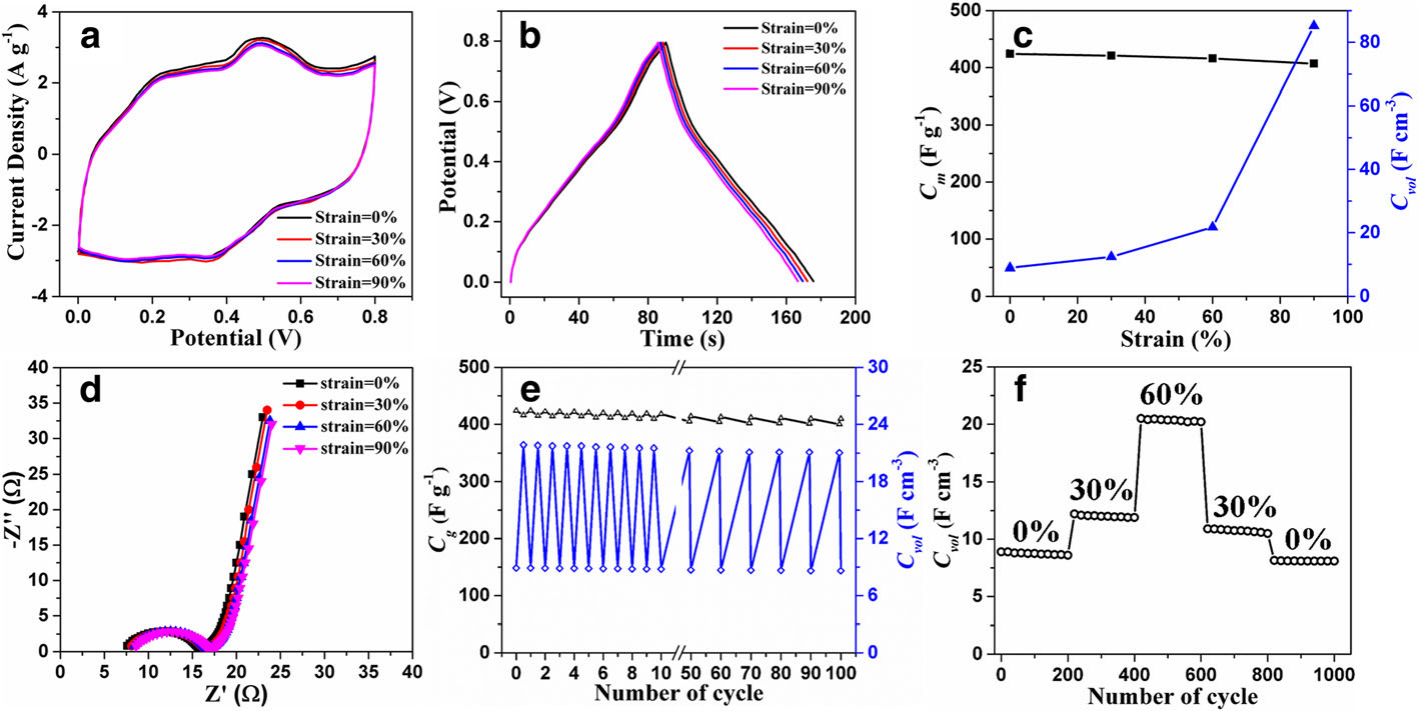
**a** CV curves, **b** GCD curves, **c** capacitive properties, and **d** Nyquist impedance plots of the SCs based on graphene/PANI-2 electrodes at various compressive strains, scan rate 20 mV s^−1^, current density 1 A g^−1^. **e** The variation of gravimetric capacitances and volumetric capacitances of graphene/PANI-2 electrodes at original state then under compressive strain of 60% for each cycle. **f** Cycle performance test for 1000 charge/discharge cycles under constant compressive strains of 0, 30 and 60%

In order to demonstrate the electrochemical performances of graphene/PANI electrodes under various compressive strains, we assembled the all-solid-state SCs. In comparison to the liquid-electrolyte-based SCs that may suffer from the possible leakage of electrolytes, the all-solid-state SCs show enhanced safety under large levels of strain [21, 32, 50]. In the graphene/PANI-2 electrodes, PVA/H_2_SO_4_ works as the solid electrolyte. The microstructure of the electrodes was observed by SEM. As shown in Additional file 1: Fig. S6, in comparison with the graphene/PANI-2 aerogel, the graphene/PANI-2 electrode with PVA/H_2_SO_4_ shows the smoother surface of cell walls. And the PVA/H_2_SO_4_ solid electrolyte tightly covered on the whole surface of the cell walls in the electrodes. As shown in Fig. 7a, the CV curves of the SCs based on graphene/PANI-2 electrodes under compression state (strain = 30%, 60%, 90%) show similar characteristics with that of the SCs at the original state (strain = 0%), indicating the good electrochemical stability of the graphene/PANI-2 electrodes under compression. The GCD curves of the SCs based on graphene/PANI-2 electrodes subjected to various compressive strains show only a little slight deviation (Fig. 7b), which verifies the compression-tolerant ability of the graphene/PANI-2 electrodes. This excellent compression-tolerant ability of the compressible electrodes arises from the synergistic effect of the two components in graphene/PANI aerogels. In graphene/PANI electrodes, the superelastic graphene aerogel provides the continuously conductive pathway and tough backbone for PANI. And the deposition of PANI not only improves the specific capacitance but also kept the high compressibility preserved. The strong interaction between PANI and graphene makes PANI tightly adhere on the cell walls during the compression-release process. The roughness mechanical performances and stable microstructure of graphene/PANI aerogels are very important for electron transport, stable conductivity, and minimizing capacitance loss. Thus, high compressible ability and structural robustness of the graphene/PANI aerogels lead to high stability of pseudo reactions and charge transfer in the electrodes at high-level compressive strains.

As shown in Fig. 7c, the SCs based on graphene/PANI-2 electrodes show the gravimetric capacitance of 424 F g^−1^ at original state and retain 96% of this value under 90% compressive strain (407 F g^−1^). The gravimetric capacitance values of graphene/PANI-2 electrodes with/without compression are higher than that of other compressible composite electrodes listed in Table 1. In addition, the volumetric capacitance of graphene/PANI-2 electrodes is dramatically improved after 60% strain, and finally reach maximum value of 85.5 F cm^−3^ at 90% strain (Fig. 7c), which is much higher than other compressible composite electrodes (Table 1). The remarkable improvement of volumetric capacitance results from almost unchanged gravimetric capacitance and significant increased density of graphene/PANI-2 electrodes under high compression. When the electrodes undergo 90% compressive strain, the density of the electrodes is 10 times the original value, and the gravimetric capacitance declines by only 4%. According to the Eq. (3), the volumetric capacitance of graphene/PANI-2 electrodes at compressive strain of 90% is 9.6 times that of them at uncompressed state.

The EIS of the SCs based on graphene/PANI-2 electrodes was also characterized (Fig. 7d). The Nyquist plots consist of a typical semicircle in the high frequency region and a straight line at low frequency. The graphene/PANI-2 electrodes show similar Nyquist plots in original and compressed states (strains of 30, 60, and 90%), verifying the compression-tolerant ability. In order to study the reversible compressibility and durability of the compressible SCs based on graphene/PANI-2 electrodes, cycle stability was demonstrated by GCD at 2 A g^−1^. Under both static (constant compressive strain) condition and dynamic (repeated compression/release) condition, there is only slight fluctuation of capacitances (Fig. 7e). For long-term durability of SCs, the compressive strains of 0, 30, and 60% are each varied at 200 charge/discharge cycles and finally, recovered to a fully relaxed state (Fig. 7f). The original volumetric capacitance of graphene/PANI-2 electrodes is preserved by 91% after 1000 charge/discharge cycles with various compressive strains. Energy density and power density are also two key factors to judge the performance of SCs. As seen from the Ragone plot (Additional file 1: Fig. S8), the maximum energy density of the SCs based on graphene/PANI-2 electrodes is 9.4 W h kg^−1^ at a power density of 0.4 kW kg^−1^. The maximum power density is 2.1 kW kg^−1^ at an energy density of 6.4 W h kg^−1^. The obtained energy density and power density are located at a moderate level among other similar all-solid-state symmetric SCs [13, 34, 51].

**Fig. 8:**
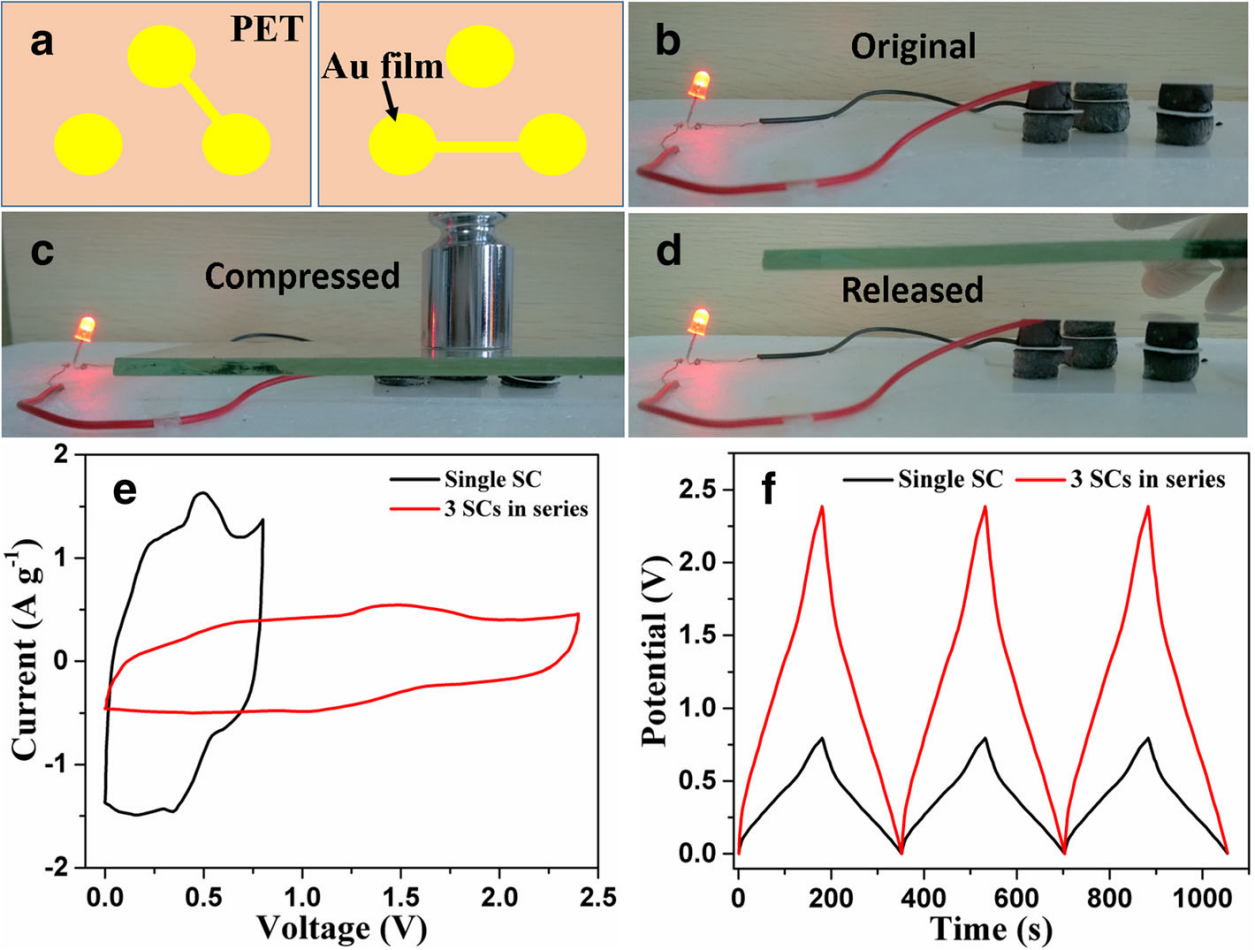
**a** Illustration of Au film patterns on PET for integrating three SCs into one unit in series. **b**–**d** Photographs of a red-light-emitting diode powered by the integrated device during the compression/release process. **e** CV curves and **f** GCD curves of single SC and integrated device. Scan rate 10 mV s^−1^, Current density 0.5 A g^−1^

The output voltage and product current of a single SC based on graphene/PANI-2 electrodes is too low to power the practical electron devices. Thus, we connected several compressible SCs either in parallel or in series to improve the output voltage or product current. As illustrated in Fig. 8a, for realizing the function of compression-tolerant ability, three compressible SCs were integrated into one unit and interconnected together in series by designing the Au film patterns on PET substrates. It can be seen in Fig. 8b–d, the resultant integrated device can light up a red-light-emitting diode and works well during the compression/release process. This integrated device was also demonstrated by CV and GCD tests. The potential window is improved from 0.8 V (for the single SC) to 2.4 V (for integrated device) in both CV and GCD curves (Fig. 3e, f). In addition, the product current (reflected by the area of CV curves) and the charge/discharge time keep unchanged for the integrated device vs individual SC, indicating that the capacitive properties of each SC in the tandem device is wall maintained.

## Conclusions

For acquiring the compressible electrodes with both high compression-tolerant ability and high capacitances, PANI was deposited into superelastic graphene aerogel by electrochemical deposition method. Different contents and uniform distribution of PANI are obtained by controlling the deposition period. Compression tests show that the recoverable compressive strain of graphene/PANI aerogels reaches 90%, indicating that the superelasticity is preserved well after the deposition of PANI. And the optimized PANI content of 63 wt%, corresponding to the specific capacitance of 713 F g^−1^ for graphene/PANI-2 aerogel, is obtained by the study in three-electrode system. The compression-tolerant ability of the graphene/PANI electrodes was demonstrated in the all-solid-state SCs. The gravimetric capacitance of the compressible graphene/PANI-2 electrodes reaches 424 F g^−1^ and retains 96% under 90% compressive strain. Resulting from the invariant of gravimetric capacitance and significant increase of density of the graphene/PANI-2 electrodes under high compression, the volumetric capacitance reaches 85.5 F cm^−3^ at 90% strain, which is far higher than other compressible composite electrodes. Furthermore, several SCs based on the graphene/PANI electrodes can be integrated and interconnected together on one chip to power the electronic devices. This work paves the way for advanced applications of SCs in the area of compressible energy-storage devices.

## Additional file

Additional file 1: Table S1. Mass content of the PANI and specific capacitance for the graphene/PANI aerogels with various deposition periods. **Table S2.** Comparison of the specific capacitance. of 3D graphene/PANI electrodes. Fig. S1 Raman spectra of GO and superelastic graphene aerogel. **Figure S2.** (a) Large-area cross-section SEM image of graphene/PANI-2 aerogel (b) and corresponding EDS element mapping images of C, (c) O and (d) N in the same area. **Figure S3.** SEM images of the graphene/PANI-4 aerogel. The PANI nanowire network cover on the surface the graphene cell walls. **Figure S4.** XRD patterns of superelastic graphene aerogel and graphene/PANI-1~3 aerogels. **Figure S5.** SEM images of graphene/PANI-2 aerogel corresponding to the (a) loading status and (b-d) unloading status. **Figure 6.** SEM images of graphene/PANI-2 electrodes covered by PVA/H SO solid electrolyte. **Figure S7.** Cycling stability of graphene/PANI-2 aerogel at a current density of 2 A g^-1^ using a three-electrode setup. **Figure S8.** (a) GCD curves of the SCs based on graphene/PANI-2 electrodes at various current densities from 1 to 5 A g^-1^. (b) Ragone plot of the SCs based on graphene/PANI-2 electrodes. (DOCX 4526 kb)
