# Supplementary material for: Graphene/Polyaniline Aerogel with Superelasticity and High Capacitance as Highly Compression-Tolerant Supercapacitor Electrode
Source: Nanoscale Res Lett. 2017 Dec 19;12:630. doi: 10.1186/s11671-017-2395-z (PMC5736521; doi:10.1186/s11671-017-2395-z)
Supplement: Additional file 1: Table S1. — Mass content of the PANI and specific capacitance for the graphene/PANI aerogels with various deposition periods. Table S2. Comparison of the specific capacitance. of 3D graphene/PANI electrodes. Fig. S1 Raman spectra of GO and superelastic graphene aerogel. Figure S2. (a) Large-area cross-section SEM image of graphene/PANI-2 aerogel (b) and corresponding EDS element mapping images of C, (c) O and (d) N in the same area. Figure S3. SEM images of the graphene/PANI-4 aerogel. The PANI nanowire network cover on the surface the graphene cell walls. Figure S4. XRD patterns of superelastic graphene aerogel and graphene/PANI-1~3 aerogels. Figure S5. SEM images of graphene/PANI-2 aerogel corresponding to the (a) loading status and (b-d) unloading status. Figure 6. SEM images of graphene/PANI-2 electrodes covered by PVA/H SO solid electrolyte. Figure S7. Cycling stability of graphene/PANI-2 aerogel at a current density of 2 A g-1 using a three-electrode setup. Figure S8. (a) GCD curves of the SCs based on graphene/PANI-2 electrodes at various current densities from 1 to 5 A g-1. (b) Ragone plot of the SCs based on graphene/PANI-2 electrodes. (DOCX 4526 kb) [file 11671_2017_2395_MOESM1_ESM.docx]

Supporting information

Graphene/polyaniline aerogel with superelasticity and high capacitance as highly compression-tolerant supercapacitor electrode

Peng Lv,* Xun Tang, Ruilin Zheng, Xiaobo Ma, Kehan Yu, Wei Wei

School of optoelectronic engineering, Nanjing University of Post & Telecommunications, Nanjing 210023, P. R. China.

Table S1 Mass content of the PANI and specific capacitance for the graphene/PANI aerogels with various deposition periods. The specific capacitances are obtained with the three-electrode system at the scan rate of 10 mV s^-1^.

| Aerogels | CV sweeping cycles | Mass content of PANI (wt%) | Specific capacitance (F g^-1^) |
| --- | --- | --- | --- |
| Graphene/PANI-1 | 100 | 48 | 485 |
| Graphene/PANI-2 | 200 | 63 | 713 |
| Graphene/PANI-3 | 300 | 79 | 609 |
| Graphene/PANI-4 | 400 | 88 | 522 |

Table S2 Comparison of the specific capacitance of 3D graphene/PANI electrodes. The capacitances are obtained with three-electrode system.

| Materials | Mass content of PANI | Specific capacitance (F g^-1^) | Test condition | Ref. |
| --- | --- | --- | --- | --- |
| Graphene/PANI hydrogel | 29 wt% | 710 | 2 A g^-1^ | [1] |
| Graphene/PANI hydrogel | - | 610 | 1 A g^-1^ | [2] |
| 3D Graphene/PANI | - | 438 | 0.5 A g^-1^ | [3] |
| Graphene/PANI foam | - | 939 | 1 A g^-1^ | [4] |
| Amino-graphene/PANI aerogel | 66 wt% | 560 | 1 A g^-1^ | [5] |
| Graphene/PANI aerogel | 52.4 wt% | 253 | 0.5 A g^-1^ | [6] |
| Graphene/PANI aerogel | 84.5 wt% | 1600 | 12 A g^-1^ | [7] |
| Graphene/PANI aerogel | 20.9 wt% | 810 | 1 A g^-1^ | [8] |
| Graphene/PANI aerogel | - | 432 | 1 A g^-1^ | [9] |
| Graphene/PANI aerogel | 57 wt% | 520 | 0.25 A g^-1^ | [10] |
| Graphene/PANI aerogel | 63 wt% | 713 | 1 A g^-1^ | This work |

References:

[1] Gao S, Zhang L, Qiao Y, Dong P, Shi J, Cao S. (2016) Electrodeposition of polyaniline on three-dimensional graphene hydrogel as a binder-free supercapacitor electrode with high power and energy densities. RSC Adv 6: 58854.

[2] Luo J, Zhong W, Zou Y, Xiong C, Yang W. (2016) Preparation of morphology-controllable polyaniline and polyaniline/graphene hydrogels for high performance binder-free supercapacitor electrodes. J Power Sources 319(1):73-81.

[3] Hong X, Zhang B, Murphy E, Zou J, Kim F. (2017) Three-dimensional reduced graphene oxide/polyaniline nanocomposite film prepared by diffusion driven layer-by-layer assembly for high-performance supercapacitors. J Power Sources 343(1): 60-66.

[4] Yu P, Zhao X, Li Y, Zhang Q. (2017) Controllable growth of polyaniline nanowire arrays on hierarchical macro/mesoporous graphene foams for high-performance flexible supercapacitors. Appl Surf Sci 393: 37-45.

[5] Mahdavi H, Kahriz PK, Gholipour-Ranjbar H, Shahalizade T. (2017) Synthesis and performance study of amino functionalized graphene aerogel grafted with polyaniline nanofibers as an efficient supercapacitor material. J Mater Sci: Mater Electron 28:4295-4305.

[6] Ye S, Feng J. (2014) Self-Assembled Three-Dimensional Hierarchical Graphene/ Polypyrrole Nanotube Hybrid Aerogel and Its Application for Supercapacitors. ACS Appl Mater Interfaces 6(12): 9671−9679

[7] Hoa NV, Quyen TTH, Hieu NV, Ngoc TQ, Thinh PV, Dat PA, Nguyen HTT. (2017) Three-dimensional reduced graphene oxide-grafted polyaniline aerogel as an active material for high performance supercapacitors. Synthetic Met 223: 192–198.

[8] Bulin C, Yu H, Ge X, Xin G, Xing R, Li R, Zhang B. (2017) Preparation and supercapacitor performance of functionalized graphene aerogel loaded with polyaniline as a freestanding electrode. J Mater Sci 52(10): 5871-5881.

[9] Yang Y, Xi Y, Li J, Wei G, Klyui NI, Han W. (2017) Flexible supercapacitors based on polyaniline arrays coated graphene aerogel electrodes. Nanoscale. Res Lett 12:394.

[10] Yang F, Xu M, Bao SJ, Wei H, Chai H. (2014) Self-assembled hierarchical graphene/polyaniline hybrid aerogels forelectrochemical capacitive energy storage. Electrochim Acta 137: 381-387.





Fig. S1 Raman spectra of GO and superelastic graphene aerogel

Raman spectra of GO and superelastic graphene aerogel display two remarkable bands at around 1345 and 1585 cm^-1^ and they are assigned to the D- and G-bands of carbon. The G-band is related to graphitic carbon and the D-band is associated with the structural defects or partially disordered structures of graphitic domains. The intensity ratio of D and G bands (I_D_/I_G_) is 0.88 in GO and increases to 1.07 in graphene aerogel, indicating the initial GO sheets were reduced to graphene by the reduction processes.


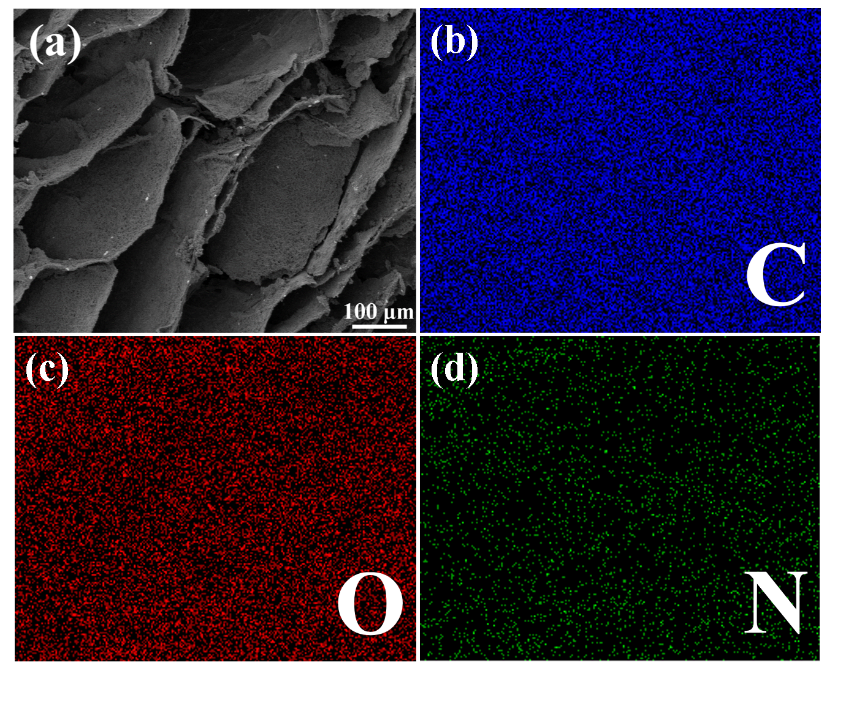


Fig. S2 (a) Large-area cross-section SEM image of graphene/PANI-2 aerogel (b) and corresponding EDS element mapping images of C, (c) O and (d) N in the same area.


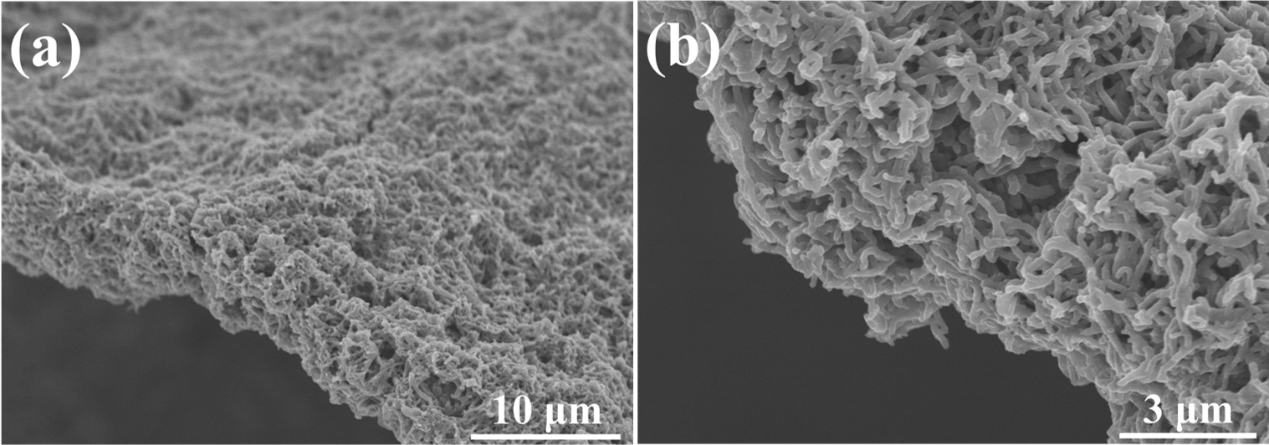


Fig. S3 SEM images of the graphene/PANI-4 aerogel. The PANI nanowire network cover on the surface the graphene cell walls.





Fig. S4 XRD patterns of superelastic graphene aerogel and graphene/PANI-1~3 aerogels


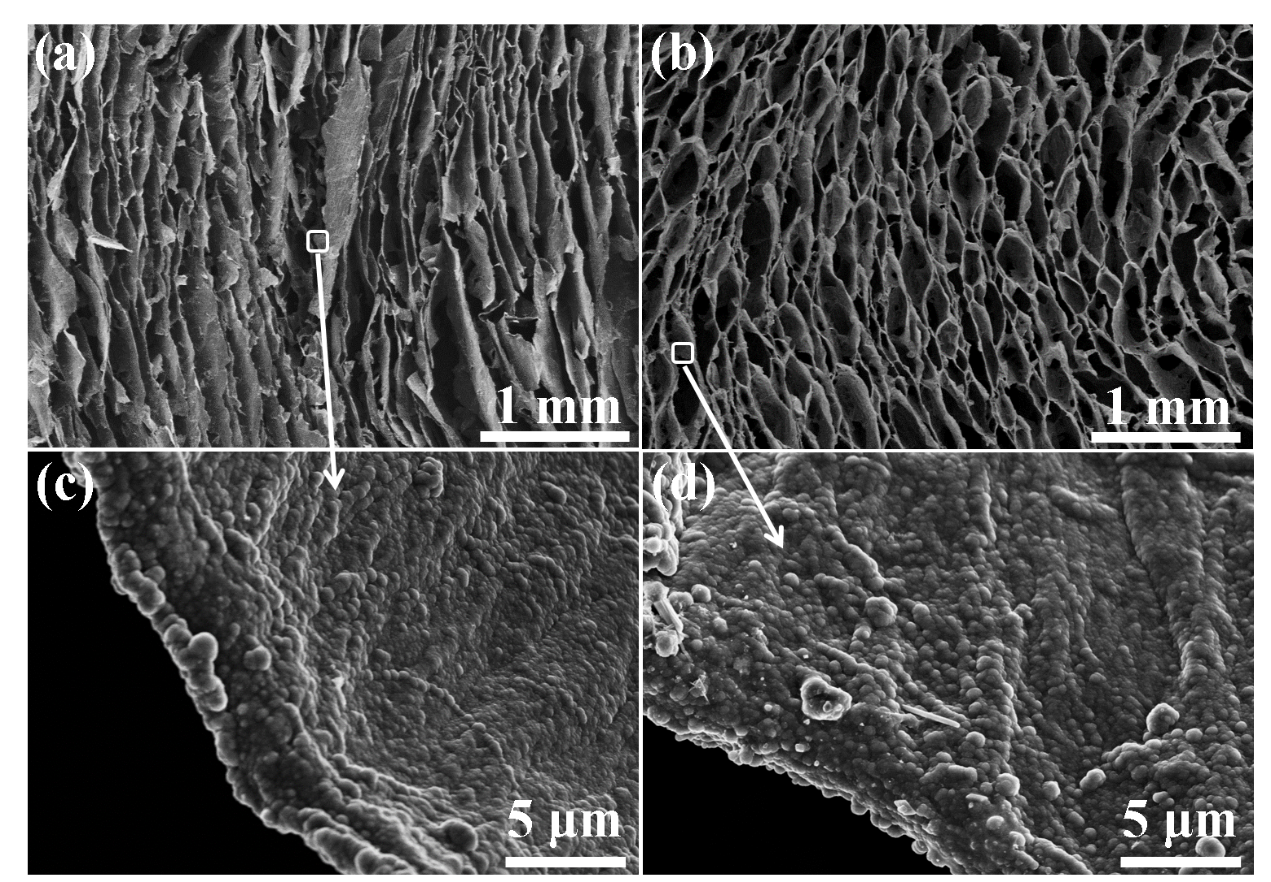


Fig. S5 SEM images of graphene/PANI-2 aerogel corresponding to the (a) loading status and (b-d) unloading status.


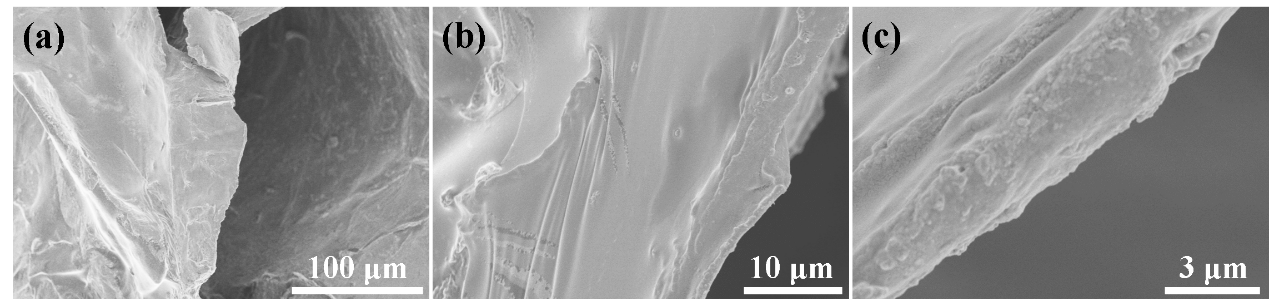


Fig. 6 SEM images of graphene/PANI-2 electrodes covered by PVA/H_2_SO_4_ solid electrolyte.





Fig. S7 Cycling stability of graphene/PANI-2 aerogel at a current density of 2 A g^-1^ using a three-electrode setup.


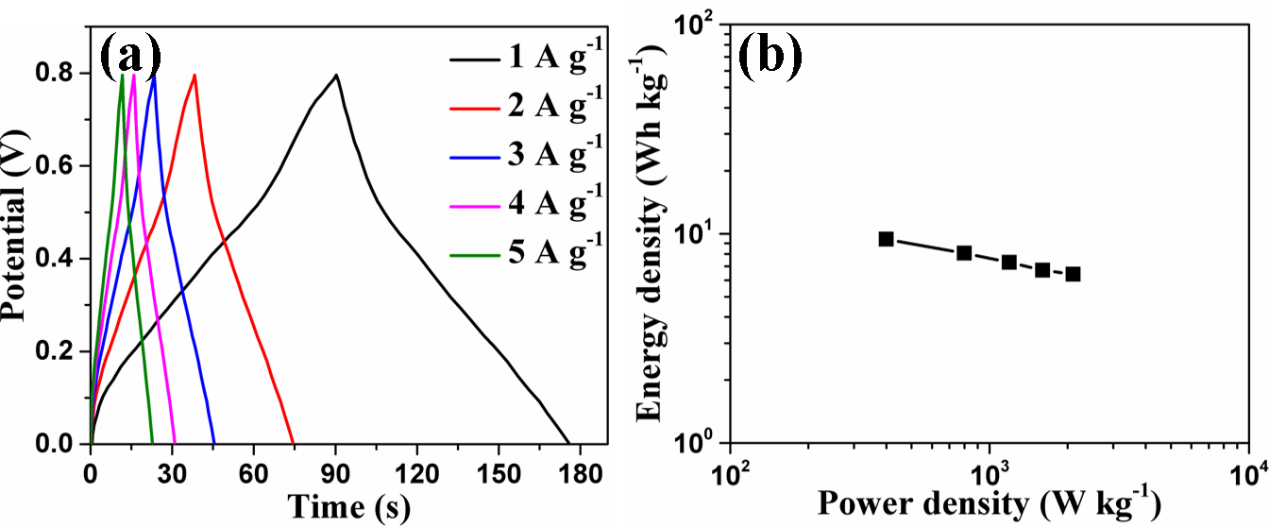


Fig S8. (a) GCD curves of the SCs based on graphene/PANI-2 electrodes at various current densities from 1 to 5 A g^-1^. (b) Ragone plot of the SCs based on graphene/PANI-2 electrodes.
